# Case Report: A case of secondary syphilis with clinical manifestations resembling granuloma annulare

**DOI:** 10.3389/fmed.2026.1899418

**Published:** 2026-08-26

**Authors:** Ting Xu, Wanyuan Chen, Yuhuan Shen, Yumei Ge

**Affiliations:** 1 Zhejiang Provincial People’s Hospital Bijie Hospital (The First People’s Hospital of Bijie), Bijie, Guizhou, China; 2 Key Laboratory for Cancer Prevention and Treatment of Guizhou Province, Bijie, Guizhou, China; 3 Zhejiang Provincial People’s Hospital (Affiliated People’s Hospital), Hangzhou Medical College, Hangzhou, Zhejiang, China; 4 Key Laboratory of Precision Medicine for Head and Neck Cancers of Zhejiang Province, Zhejiang Provincial People’s Hospital, Hangzhou, Zhejiang, China; 5 Laboratory Medicine Center, Department of Clinical Laboratory, Zhejiang Provincial People’s Hospital, Affiliated People’s Hospital, Hangzhou Medical College, Hangzhou, Zhejiang, China

**Keywords:** granulomatous inflammation, histopathology, skin, syphilis, *Treponema pallidum*

## Abstract

Syphilis is caused by *Treponema pallidum* and progresses through different clinical stages, with a wide range of clinical manifestations. We report a case of secondary syphilis in which the patient presented with diffuse granuloma annulare–like lesions involving the neck, chest, abdomen, back, and upper extremities. Clinically, the lesions manifested as annular plaques with raised erythematous borders, accompanied by central ulceration and necrosis. The diagnosis of secondary syphilis was confirmed by skin biopsy and serological examination. Following treatment with benzathine penicillin G, the non-treponemal test (TRUST) became negative 7 months after therapy. The patient responded well to intravenous penicillin treatment, highlighting the importance of early detection and timely therapy in managing syphilis with atypical histopathological features. In recent years, the incidence of syphilis has continued to increase, underscoring the importance of recognizing this clinicopathological presentation. Routine syphilis screening is essential for accurate diagnosis and appropriate treatment.

## Introduction

Syphilis is an infection caused by *Treponema pallidum* and is most commonly transmitted through sexual contact with an infected individual (1). Syphilis progresses from a primary stage, which often goes unnoticed, to a secondary stage that typically occurs 6–11 weeks after the initial exposure. Secondary syphilis presents with a wide spectrum of cutaneous manifestations, including lesions on the trunk, face, and genitalia, as well as the characteristic papulosquamous eruptions on the palms and soles.

Nearly one-third of untreated patients with primary or secondary syphilis may develop late manifestations, such as cutaneous syphilis, neurosyphilis, or cardiovascular syphilis. Cutaneous tertiary syphilis accounts for approximately 16% of cases (2). According to data from the World Health Organization, there were 7.1 million new cases among adults worldwide in 2020, underscoring the importance for healthcare providers to remain aware of this alarming trend and the evolving epidemiology of sexually transmitted infections (3).

The cutaneous manifestations of tertiary syphilis include nodular serpiginous syphilis, nodular ulcerative syphilis, and gummatous syphilis (4). Given the recent increase in syphilis cases, recognition of clinical and histopathological changes is crucial to facilitate timely diagnosis and treatment. In this report, we present a case of secondary syphilis with clinical features resembling granuloma annulare.

## Case demonstration

**Figure 1:**
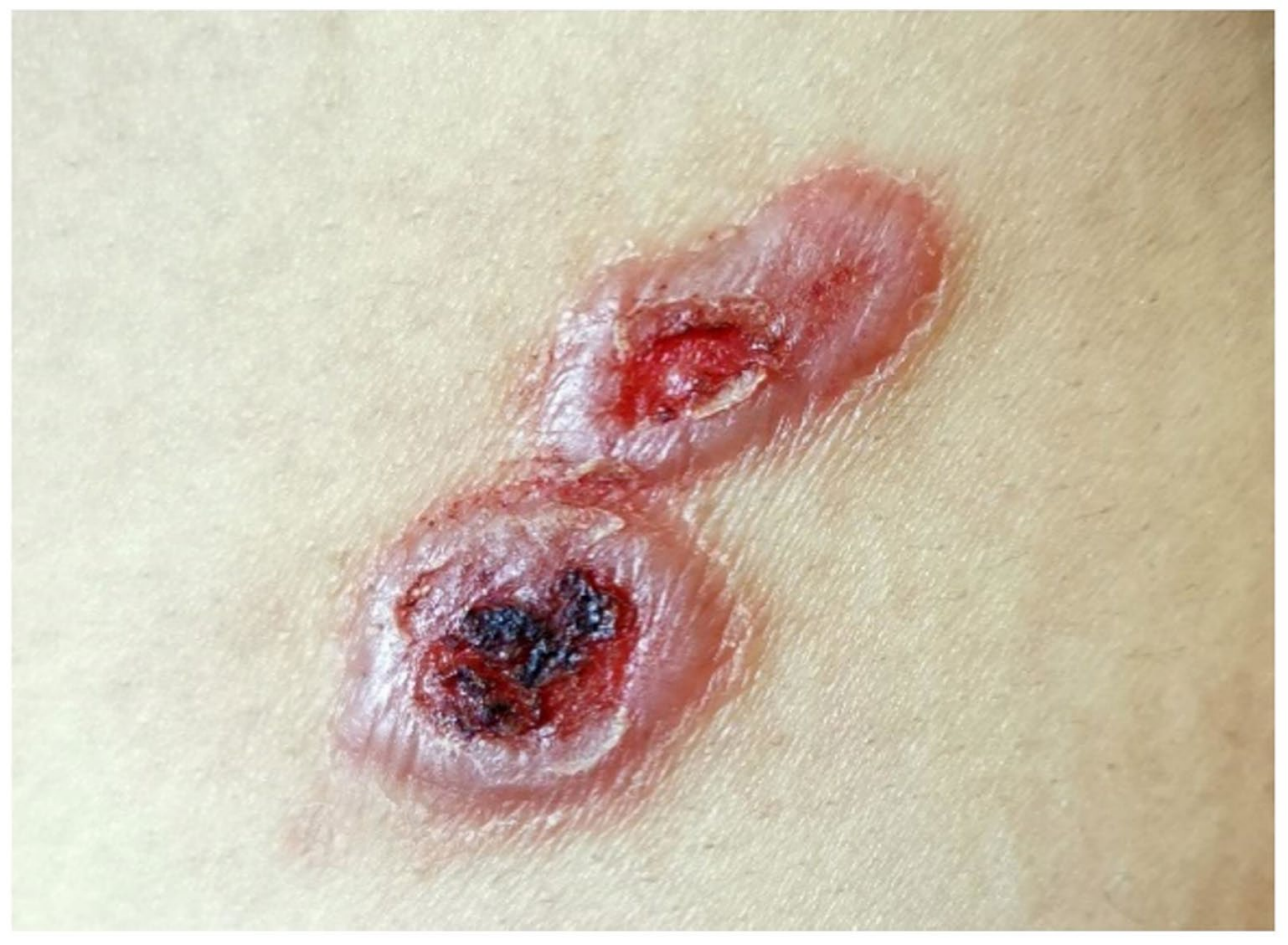
Abdominal lesions of the patient. Oval pink erythematous plaques with raised, deep, and irregular borders, exhibiting central ulceration covered with black scabs, necrotic debris, and hemorrhagic exudate.

A 22-year-old female with no prior medical history presented with annular erythema on the neck that appeared 2 months ago and gradually spread to the chest, upper limbs, back, and abdomen, accompanied by pruritus. Physical examination revealed scattered annular erythematous plaques with raised borders and central necrosis (Figure 1). Serological tests were positive for both treponemal-specific antibodies (Tp-Ab) and non-treponemal antibodies (TRUST), with a TRUST titer of 1:32.

**Figure 2:**
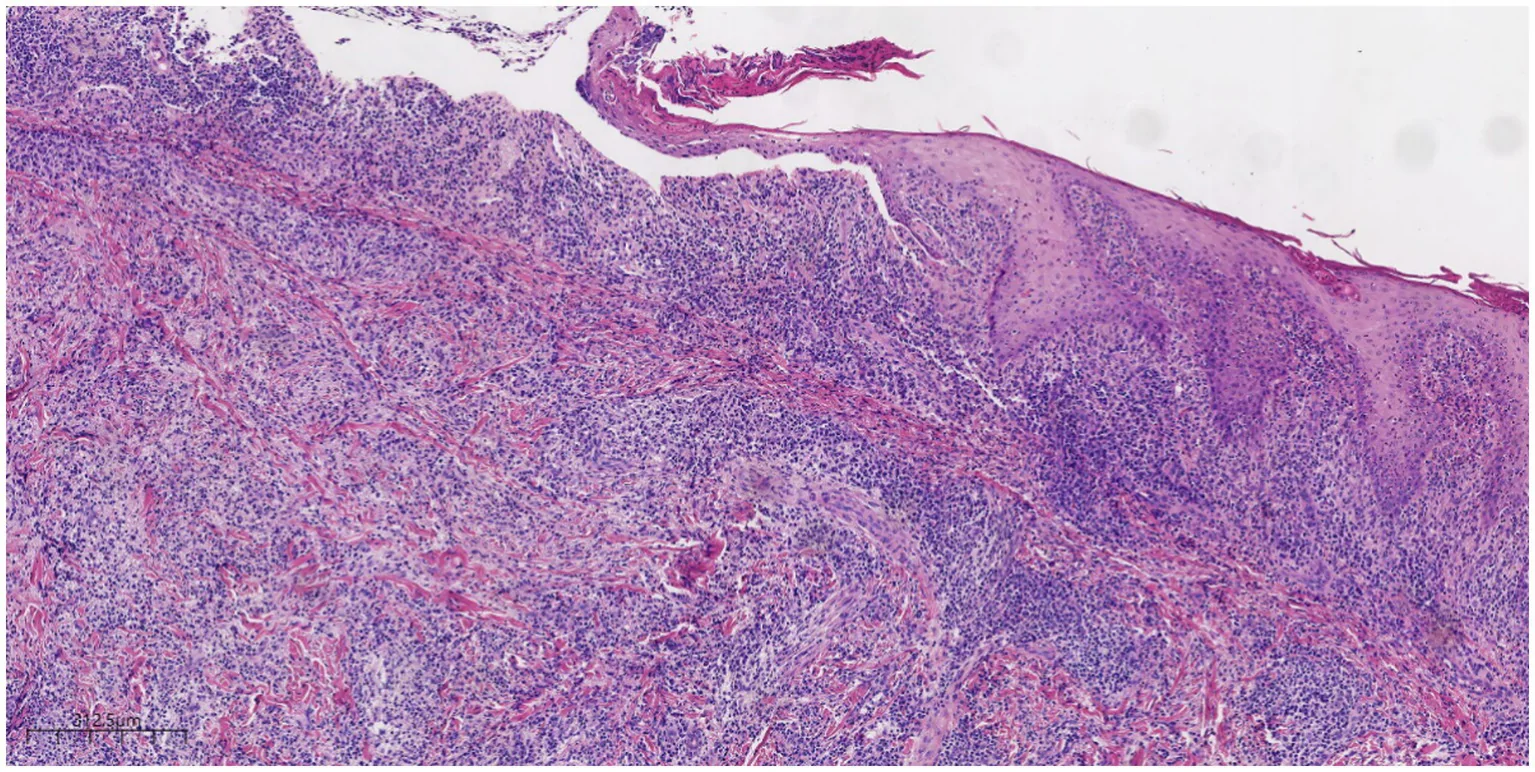
Under low-power magnification, focal epidermal ulceration was observed, with areas of hyperkeratosis and parakeratosis. The epidermis and dermis showed dense infiltration of lymphocytes and plasma cells (H&E × 40).

**Figure 3:**
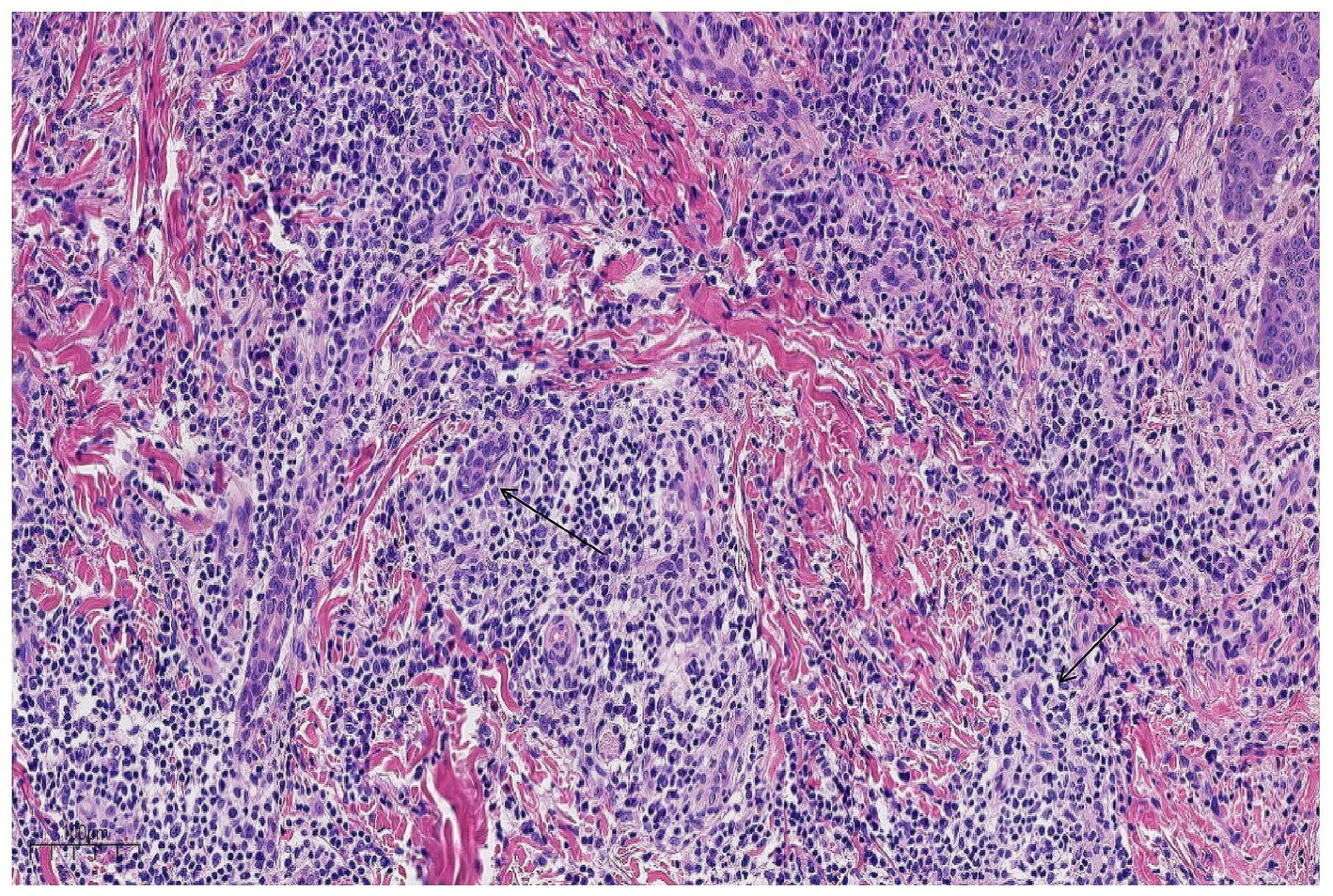
Small vessel occlusion with surrounding inflammation observed under medium-power magnification (H&E × 100, indicated by black arrows).

**Figure 4:**
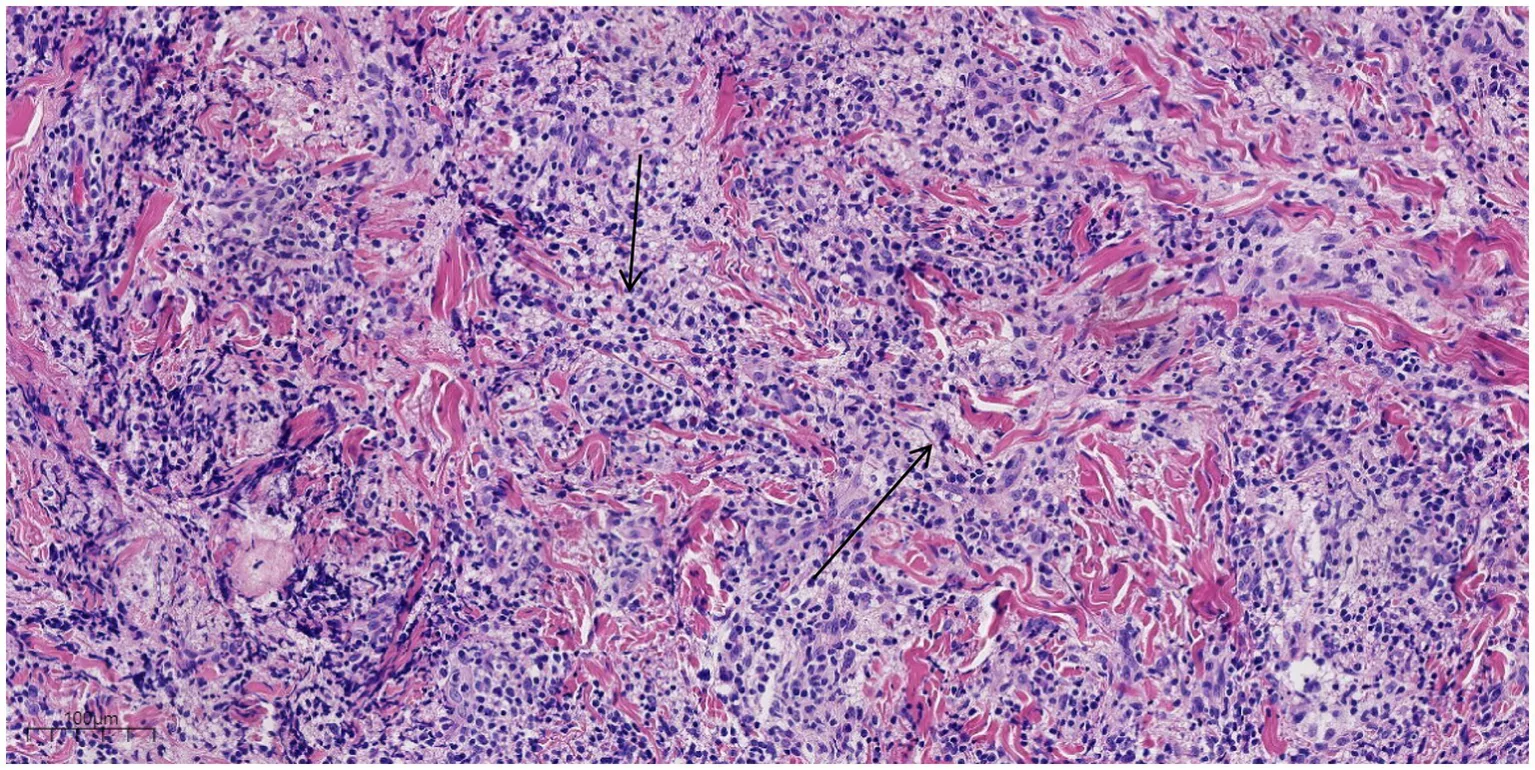
Fragmentation of elastic fibers observed under medium-power magnification (H&E × 100, indicated by black arrows).

**Figure 5:**
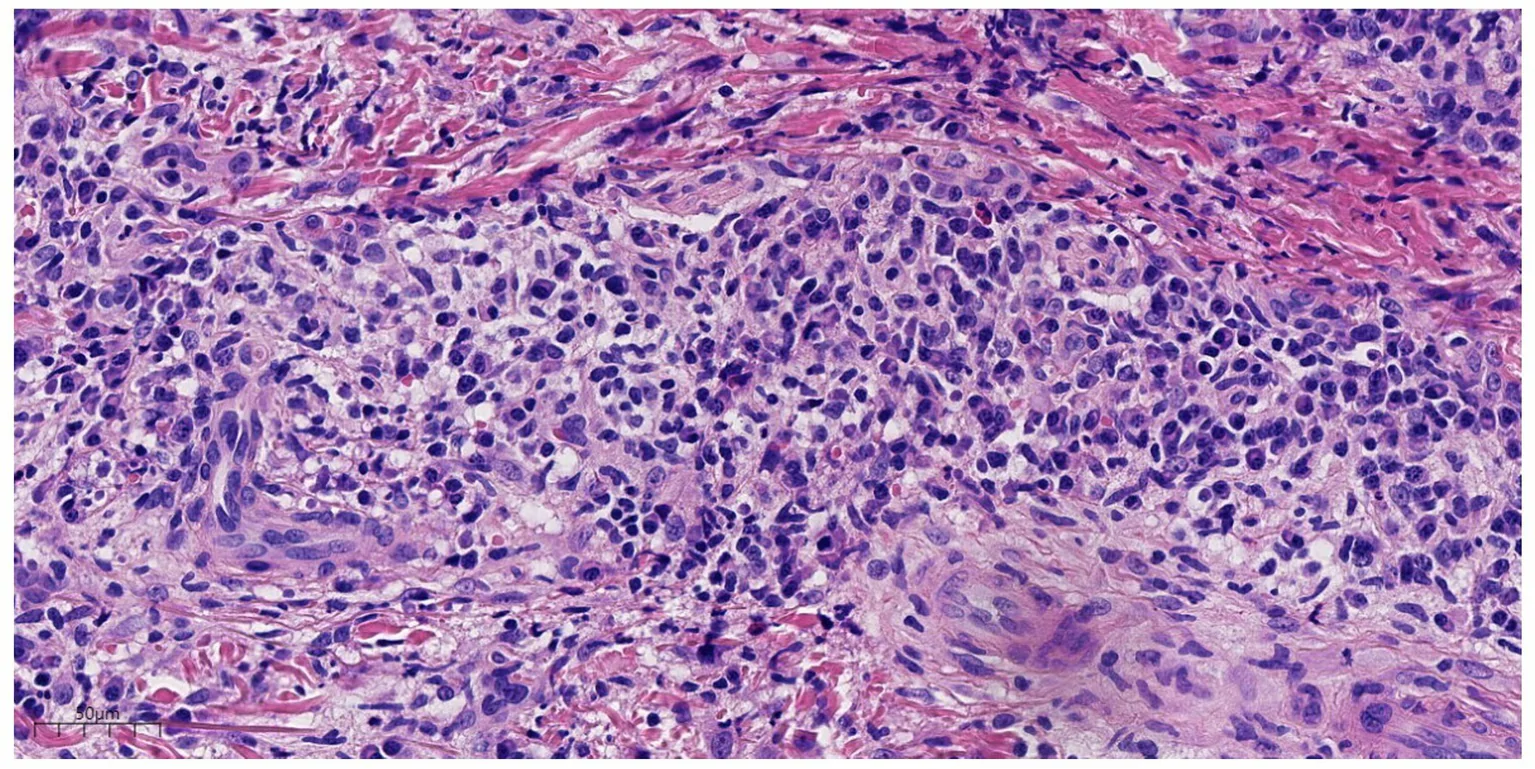
Under high-power magnification, dense inflammatory cell infiltration was observed, predominantly composed of plasma cells (H&E × 200).

The lesions progressively enlarged, forming granuloma annulare–like patterns, with central necrosis emerging. A skin biopsy was performed on the left abdomen for histopathological examination. Under low-power magnification, focal epidermal ulceration was observed, with areas of hyperkeratosis and parakeratosis. The epidermis and dermis showed dense infiltration of lymphocytes and plasma cells (Figure 2) under medium-power magnification, Small vessel occlusion with surrounding inflammation observed under medium-power magnification (Figure 3), Fragmentation of elastic fibers observed under medium-power magnification (Figure 4). Under high-power magnification, dense inflammatory cell infiltration was observed, predominantly composed of plasma cells (Figure 5). Combined with the clinical history, the histopathological changes were consistent with syphilitic infection.

Upon further history taking, the patient reported unprotected sexual contact with a syphilis-positive partner. The patient was diagnosed with secondary syphilis with cutaneous manifestations. She received intramuscular injections of benzathine penicillin G as follows: on November 11, 2020, 1.2 million IU + 2.4 million IU split between both gluteal muscles; November 18, 2020, 1.2 million IU + 2.4 million IU split between both gluteal muscles; 1 week later, 1.2 million IU + 2.4 million IU split between both gluteal muscles; December 3, 2020, 1.2 million IU + 2.4 million IU split between both gluteal muscles; and December 16, 2020, 1.2 million IU + 2.4 million IU split between both gluteal muscles. The total cumulative dose of benzathine penicillin G was 12 million IU (given as five doses of 2.4 million IU each, at weekly intervals). Given the late stage of disease, multiple high-dose penicillin injections were administered to treat secondary syphilis and its cutaneous manifestations.

**Figure 6:**
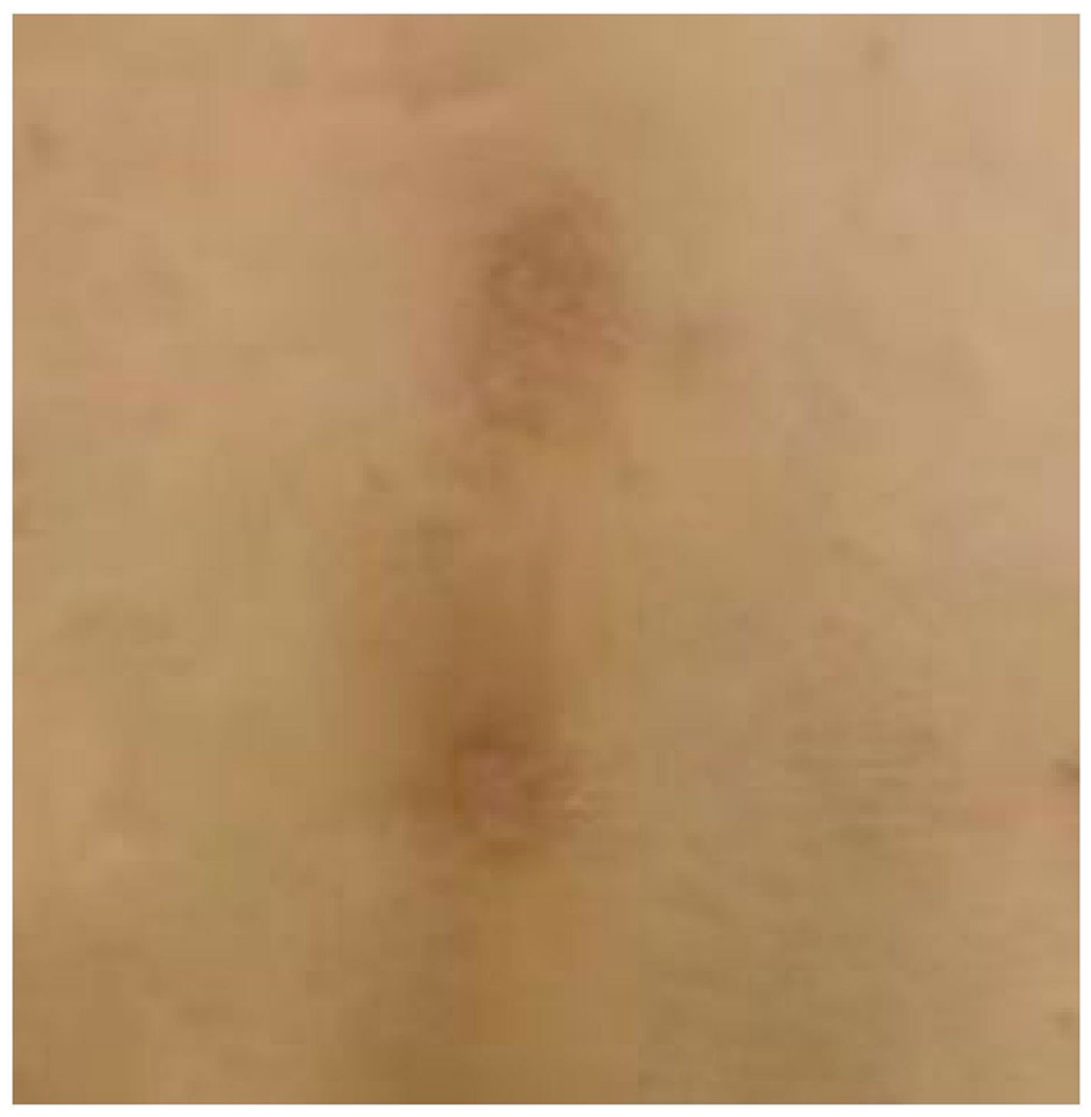
Post-treatment lesions on the patient’s back. Erythematous or granulomatous lesions have healed, leaving hyperpigmented scars.

The patient responded well to treatment. After 3 weeks, the TRUST titer decreased to 1:2, and 7 months later, the TRUST test became negative. The lesions healed with residual post-inflammatory hyperpigmented scars (Figure 6).

## Discussion

Secondary syphilis typically presents with generalized and symmetric skin rashes (5). In our case, the patient exhibited diffuse, asymmetric, annular granulomatous lesions. The presence of multiple widespread granuloma annulare-like lesions posed a diagnostic challenge, requiring differentiation from sarcoidosis, lichen planus, necrobiosis lipoidica, and urticaria pigmentosa.

Sarcoidosis shows reddish-brown papules/plaques on the face and extensor surfaces, often with hilar lymphadenopathy; histologically, it features “naked” non-caseating granulomas with sparse lymphocytes and rare plasma cells (6). Lichen planus presents as violaceous, pruritic, polygonal papules with Wickham’s striae on the flexor wrists and oral mucosa; histology shows hyperkeratosis, liquefactive degeneration of basal cells, and a band-like lymphocytic infiltrate without plasma cell predominance (7). Necrobiosis lipoidica typically affects pretibial areas in diabetic patients, presenting as yellowish-brown atrophic plaques; histology reveals extensive collagen necrobiosis with palisaded granulomas and fibrosis, but plasma cells are inconspicuous (8). Urticaria pigmentosa features brownish macules/papules with Darier’s sign, predominantly in children; histology shows mast cell aggregates in the superficial dermis, with no plasma cell infiltration (9).

Previous reports indicate that most previously documented cases of secondary granulomatous syphilis lesions persist for more than 4 weeks, which is consistent with our patient’s course, as the interval from onset to diagnosis was 2 months.

Histopathological features of syphilis vary according to disease stage (10). In primary syphilis, histology shows epidermal erosion or ulceration, dermal infiltration of lymphocytes and plasma cells, endothelial cell swelling and proliferation around vessels, and spirochetes located at the dermo-epidermal junction and perivascular areas. Secondary syphilis demonstrates highly variable and overlapping inflammatory patterns, including a band-like infiltrate in the upper dermis, perivascular and periadnexal infiltrates extending into mid- and deep-dermis, predominantly plasma cells and lymphocytes, with occasional histiocytes and granulomatous infiltrates, stromal inflammation, endothelial cell swelling, irregular epidermal rete ridges, and abundant spirochetes in the epidermis. Tertiary syphilis exhibits dense lymphocyte and plasma cell infiltration extending into the dermis and subcutaneous fat, with plasma cells sometimes sparse or absent, conspicuous caseating granulomas, and multinucleated giant cells (5).

Several cases of granuloma annulare-like syphilis have been reported. Sezer et al. (10) described an interstitial granuloma annulare-like pattern with admixed plasma cells, while our case showed annular plaques with central necrosis. Liu et al. (1) reported annular elastolytic giant cell granuloma associated with syphilis, whereas our case lacked elastophagocytosis and featured dense perivascular plasma cell infiltration. Our findings broaden the morphological spectrum of this entity.

Guarino et al. (11) reviewed 55 cases of oropharyngeal syphilis, finding tonsillar predominance (71%) and ulcerative lesions as the main presentation in primary (56%) and secondary (36%) syphilis, with malignancies as a key mimicker. Although our patient lacked oropharyngeal involvement, these findings highlight syphilis’s variable presentations and the need to consider it in atypical inflammatory lesions, especially given its rising global incidence.

In our case, histopathology showed focal epidermal ulceration and dense infiltration of plasma cells and lymphocytes around superficial dermal blood vessels and adnexal structures. Histological detection of spirochetes is commonly performed using silver impregnation techniques, such as Warthin–Starry and Levaditi staining. However, one study reported that immunohistochemistry offers higher sensitivity for detecting spirochetes in biopsy specimens from primary and secondary mucocutaneous lesions (12). For the diagnosis of primary or secondary syphilis, direct detection of *T. pallidum* from lesion exudate or tissue remains the preferred confirmatory method. Secondary granulomatous inflammation is usually associated with sparse or absent spirochetes. Although no specific staining or immunohistochemical detection was performed in our case, the diagnosis was supported by the characteristic histopathological changes, positive serology, and favorable therapeutic response to penicillin.

Accurate staging of syphilis is crucial for clinical management. This relies on detailed sexual and medical history, physical examination, and patient serological testing (13). In China, all hospitalized patients undergo initial syphilis screening, and positive cases are confirmed with repeat testing. Serological testing for *T. pallidum* is the preferred method for screening, diagnosis, and monitoring treatment response (13). Confirmed patients, as well as their sexual contacts, should be evaluated and treated accordingly. Benzathine penicillin G is the first-line treatment for syphilis (5).

In our case, the diagnosis of secondary syphilis was definitive. Following four courses of benzathine penicillin G, the non-treponemal antibody titers became negative, and the cutaneous lesions healed. Final management recommendations included counseling the patient regarding potential treatment response and monitoring both clinical and serological outcomes post-therapy.

## Conclusion

Our case demonstrates a unique presentation of secondary granulomatous syphilitic cutaneous lesions with distinctive clinicopathological features. The presence of granulomas poses a significant diagnostic challenge for clinicians and pathologists; however, histopathological examination combined with serological testing supported the diagnosis of syphilis. Accurate diagnosis enabled appropriate treatment, resulting in a favorable outcome for the patient.

## Data Availability

The original contributions presented in the study are included in the article/supplementary material, further inquiries can be directed to the corresponding author.
